# Atmospheric Circulation Patterns Associated with Extreme United States Floods Identified via Machine Learning

**DOI:** 10.1038/s41598-019-43496-w

**Published:** 2019-05-09

**Authors:** Katherine E. Schlef, Hamid Moradkhani, Upmanu Lall

**Affiliations:** 1 Consortium of Universities for the Advancement of Hydrologic Science, Inc., Cambridge, MA USA; 2 Department of Civil, Construction and Environmental Engineering, University of Alabama, Tuscaloosa, AL USA; 3 Department of Earth and Environmental Engineering, Columbia University, New York, NY USA

**Keywords:** Natural hazards, Atmospheric science

## Abstract

The massive socioeconomic impacts engendered by extreme floods provides a clear motivation for improved understanding of flood drivers. We use self-organizing maps, a type of artificial neural network, to perform unsupervised clustering of climate reanalysis data to identify synoptic-scale atmospheric circulation patterns associated with extreme floods across the United States. We subsequently assess the flood characteristics (e.g., frequency, spatial domain, event size, and seasonality) specific to each circulation pattern. To supplement this analysis, we have developed an interactive website with detailed information for every flood of record. We identify four primary categories of circulation patterns: tropical moisture exports, tropical cyclones, atmospheric lows or troughs, and melting snow. We find that large flood events are generally caused by tropical moisture exports (tropical cyclones) in the western and central (eastern) United States. We identify regions where extreme floods regularly occur outside the normal flood season (e.g., the Sierra Nevada Mountains due to tropical moisture exports) and regions where multiple extreme flood events can occur within a single year (e.g., the Atlantic seaboard due to tropical cyclones and atmospheric lows or troughs). These results provide the first machine-learning based near-continental scale identification of atmospheric circulation patterns associated with extreme floods with valuable insights for flood risk management.

## Introduction

Flooding is an ever-present socio-economic risk that is likely to increase in the future under climate change and human development^1–3^. This risk has led to a variety of studies on the natural and anthropogenic causes of floods. Enhancing the predictability of floods through improved understanding of the causal natural mechanisms of flooding requires both a watershed perspective (i.e., evaluation of the status of the catchment, such as land cover, slope, aspect, morphology, initial conditions, and the nature of precipitation inputs) and an atmospheric perspective (i.e., evaluation of synoptic-scale atmosphere circulation patterns and the predictability of precipitation inputs).

Based on studies at the watershed scale, the proximate natural causes of hydrologic floods can be classified as single-day rainfall that rapidly exceeds infiltration capacity, multi-day to week-long rainfall that exceeds soil moisture holding capacity, several-day rainfall that results in a combination of the mechanisms associated with single- and multi-day rainfall, and snowmelt or rain-on-snow^4,5^. Proximate flood causes are driven by ultimate causes at various spatiotemporal scales, such as (extra)tropical cyclones, sea surface temperature anomalies, and preferred ridge and trough positions^6^. The importance of ultimate causes in generating flood events has been substantiated by global-scale studies of the correlation between floods and well-known climate patterns such as the El Niño-Southern Oscillation^7,8^, continental-scale studies which identify atmospheric circulation patterns associated with floods or further investigate the correlation between climate indices and floods^9,10^, and the myriad of regional- to local-scale studies that establish teleconnections between floods and oceanic-atmospheric patterns^11–13^.

While the natural causes of annual floods have been well-studied and summarized for each state in the United States^14^; surprisingly, assessment of the natural causes of extreme (e.g., return period of at least 10 years) floods is often limited to event- and regional-scale analyses. For example, studies of this type have analyzed the June 2008 floods in Iowa^15^, floods in the Ohio River basin and the Southwestern United States with return period greater than 10 years^16,17^, and floods in southeastern Australia causing record river heights^18^. Given the extensive damages caused by extreme floods, it is imperative to develop a unified understanding of the natural causes of extreme floods at a near-continental scale. In particular, focusing on atmospheric circulation patterns leading to heavy rainfall, which is an important factor in nearly all except snowmelt-driven extreme floods, can be especially used to inform continental-scale modeling and forecasting efforts, including the one underway with the National Water Model^19^.

We use self-organizing maps (SOMs), a type of artificial neural network which performs unsupervised clustering, to identify dominant atmospheric circulation patterns (i.e., synoptic-scale ultimate causes of extreme precipitation, that we refer to as *circulation patterns* or simply *patterns*) associated with extreme floods across the United States. We assess the characteristics of floods associated with each circulation pattern, including the occurrence frequency, spatial domain, size of events, and seasonality. This evaluation of the atmospheric circulation patterns associated with extreme floods at the near-continental scale provides a first-order basis for developing an understanding of how future flood risk may change through the lens of potential changes in atmospheric circulation patterns.

## Results

### Circulation patterns associated with extreme floods

**Figure 1:**
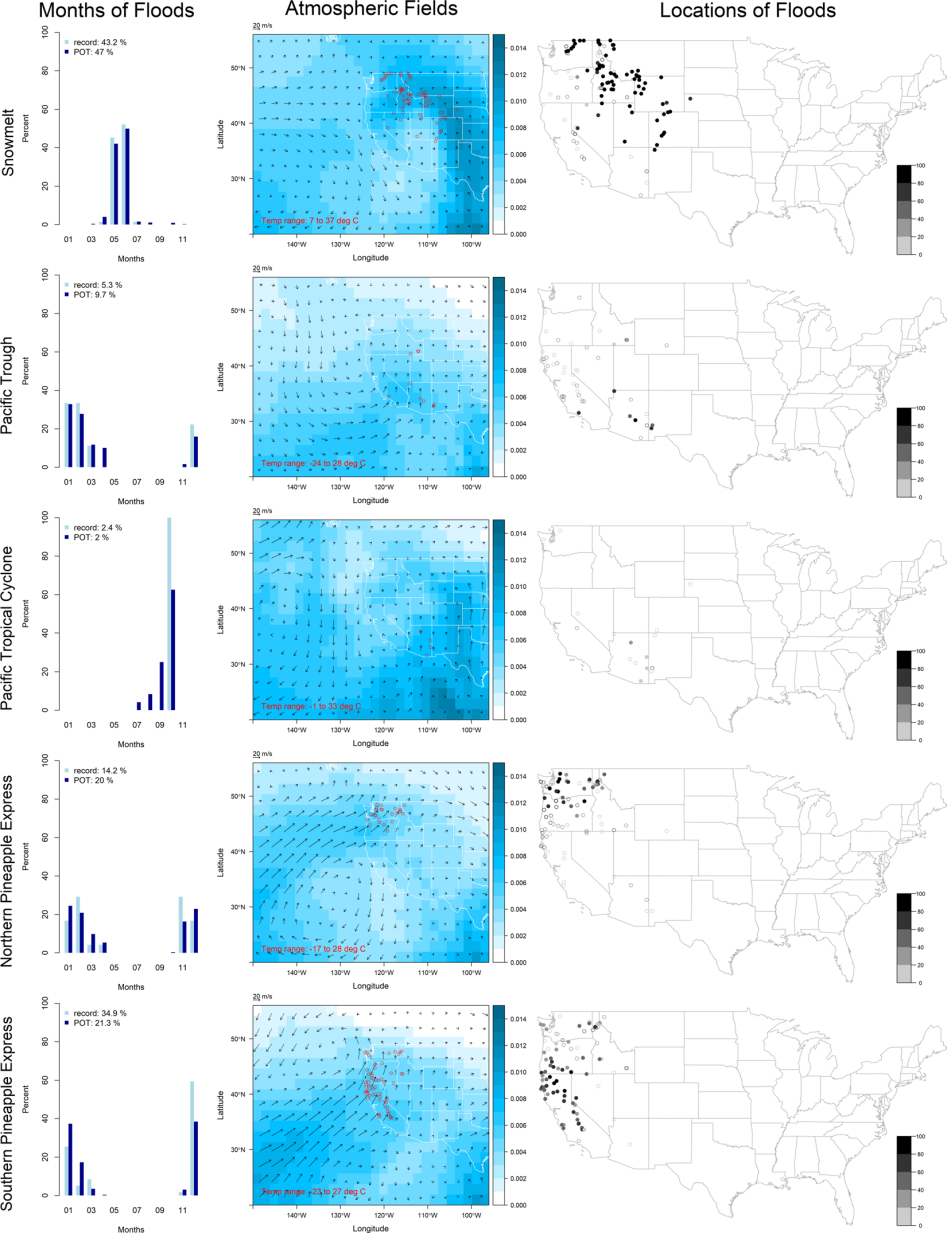
Circulation patterns in the West region. Each row represents a unique pattern. Column one shows the percent of record or POT floods occurring in each month for that pattern (the legend provides the percent of record or POT floods assigned to that pattern relative to all patterns in the region). Column two shows the wind vectors (multiplied by two for plotting, units m/s) and the specific humidity field (units kg/kg, magnitude indicated by the color bar) representative of the cluster (the text indicates the temperature range, and the circles are locations of record floods in that cluster); for visualization purposes, the inverse of normalization was applied to the atmospheric fields obtained from the SOM. Column three shows the location of record and POT floods (dots and circles, respectively) in that cluster (the color bar indicates the percent of POT floods assigned to that cluster).

The circulation patterns associated with extreme floods in the contiguous United States are grouped into three regions: West, Central and East. The West region (Fig. 1, Table 1) is represented by a 2 × 3 SOM which includes gages in hydrologic unit codes (HUCs) 14 through 18 and any gages in HUC 10 at elevation higher than 4,000 feet, for a total of 169 record floods and 1,223 peaks-over-threshold (POT) floods. The West region has a longitude-latitude domain of 20°–56°N and 150°–96°W for the atmospheric fields. Note that only five, not six, patterns are shown (see Supplemental Material for explanation). West region circulation patterns are *snowmelt*, *Pacific trough*, *Pacific tropical cyclone*, and *northern* and *southern pineapple express*.

**Table 1:** Circulation patterns in the West region.

SOM Code Number	Pattern Name (Abbreviation)	Number Record (POT) Floods	Timing	Atmospheric Signature	Primary Location of Influence	Localized Mechanisms
1	Snowmelt (Snowmelt)	73 (575)	May–Jun	Warm temperatures, relatively weak winds, high specific humidity on the eastern side of the Rocky Mountains	High elevations in Rocky Mountains and elsewhere	Snowmelt, may be augmented by heavy rain^42,43^
3	Pacific Trough (Pacific_Tr)	9 (119)	Dec–Mar	Cold temperatures and low specific humidity in the north, clear trough pattern off the West coast that directs central Pacific moisture into the Southwest which may form a tropical moisture export^17,44^	Southwest, and California, Nevada, and Idaho	Heavy or prolonged rain, may be augmented by orographic lift, snowmelt, and runoff over frozen ground^45,46^
4	Pacific Tropical Cyclone (Pacific_TC)	4 (24)	Sep–Oct	Relatively warm temperatures, the North Pacific High centered at 40°N and 145°W and northward moisture transport from the Great Plains low level jet at 100°W indicate a typical North American Monsoon pattern^47,48^	Arizona	Heavy rain from a tropical cyclone or its remnants combined with other weather systems^49,50^
5	Northern Pineapple Express (North_PE)	24 (245)	Nov–Apr	Cold temperatures and a narrow band of south-westerly winds with high specific humidity steered northward by the North Pacific High centered at 30°N and 130°W, evidence of a tropical moisture export^31,51^	Washington and northern Oregon	Heavy warm rain and rain-on-snow at higher elevations^52,53^
2,6	Southern Pineapple Express (South_PE)	59 (260)	Dec–Mar	Cold temperatures and a narrow band of strong south-westerly winds with high specific humidity aligned directly from the central Pacific towards the West coast, evidence of a tropical moisture export^31,51^	California and southern Oregon	Heavy warm rain and rain-on-snow at higher elevations^21,54^

**Figure 2:**
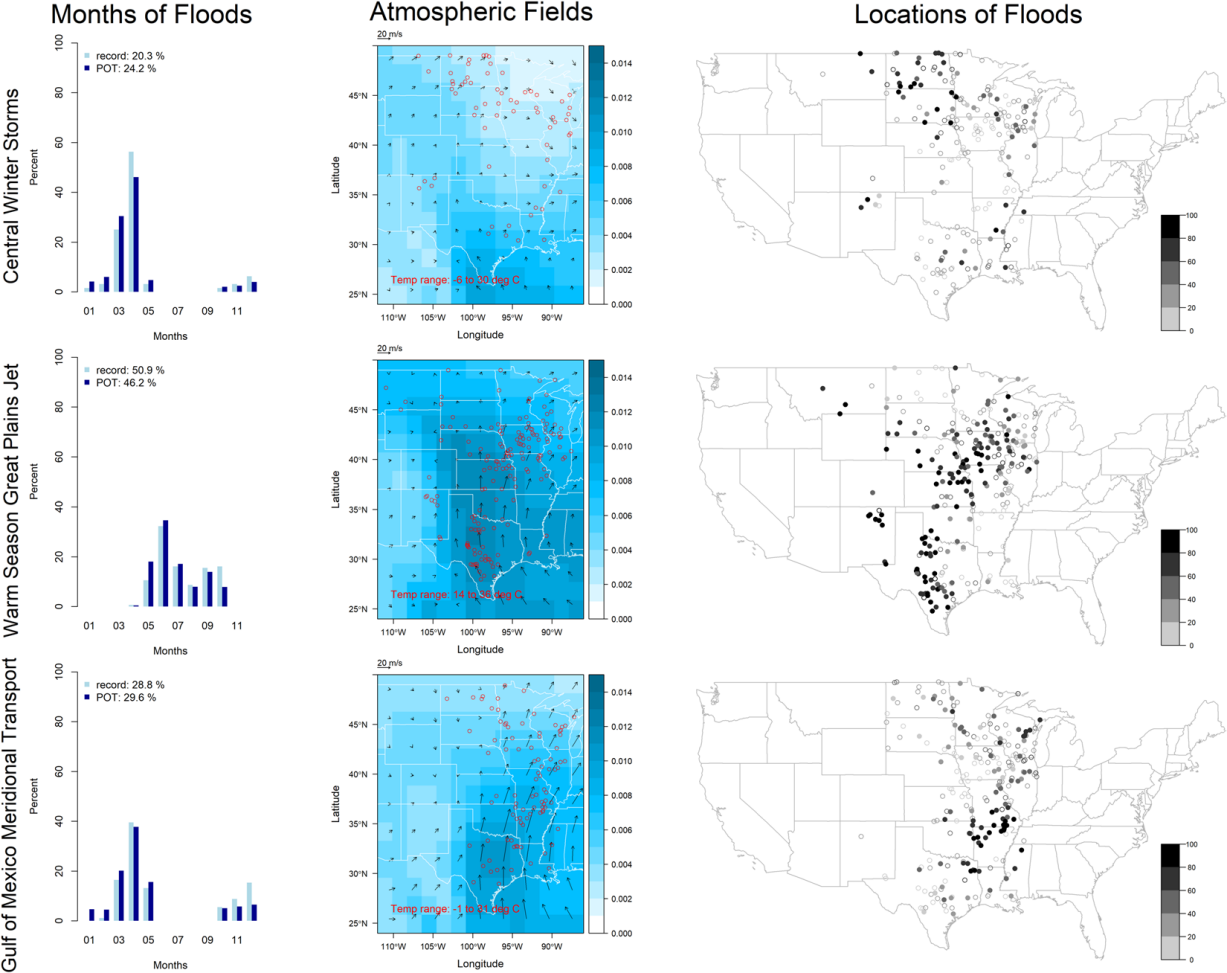
Circulation patterns in the Central region (see Fig. 1 for explanation).

The Central region (Fig. 2, Table 2) is represented by a 1 × 3 SOM which includes gages in HUCs 7 through 9 and 11 through 12, any gages in HUC 10 at elevation less than 4,000 feet, and any gages in HUC 4 west of 87°W, for a total of 316 record floods and 2,183 POT floods. The Central region has a longitude-latitude domain of 24°–50°N and 112°–86°W for the atmospheric fields. Central region circulation patterns are *central winter storm*, *warm season Great Plains jet*, and *Gulf of Mexico meridional transport*.

**Table 2:** Circulation patterns in the Central region.

SOM Code Number	Pattern Name (Abbreviation)	Number Record (POT) Floods	Timing	Atmospheric Signature	Primary Location of Influence	Localized Mechanisms
1	Central Winter Storm (Central_WS)	64 (529)	Oct–May	Cold temperatures, weak winds from the north, low specific humidity (note that some individual events show evidence of low pressure systems)^55,56^	Northern and southern Midwest	Rain-on-snow in the north and heavy rains in the south^45,57^
2	Warm Season Great Plains Jet (Warm_GPJ)	161 (1,008)	Apr–Oct	Warm temperatures, strong southerly winds from the Gulf of Mexico confined to a narrow region, high specific humidity, evidence of a tropical moisture export, colloquially known as the Maya Express^31,58^	Throughout the Midwest (more concentrated in western portion)	Heavy rainfall, may be augmented by wet antecedent conditions (and delayed snowmelt in Montana)^15,59^
3	Gulf of Mexico Meridional Transport (central) (GofM_MT(c))	91 (646)	Oct–May	Cold temperatures, strong southerly winds from the Gulf of Mexico confined to a narrow region, relatively high specific humidity, evidence of a tropical moisture export^16,58,60,61^	Throughout the Midwest (more concentrated in eastern portion)	Heavy rainfall, may be augmented by rapid snowmelt^22,45^

**Figure 3:**
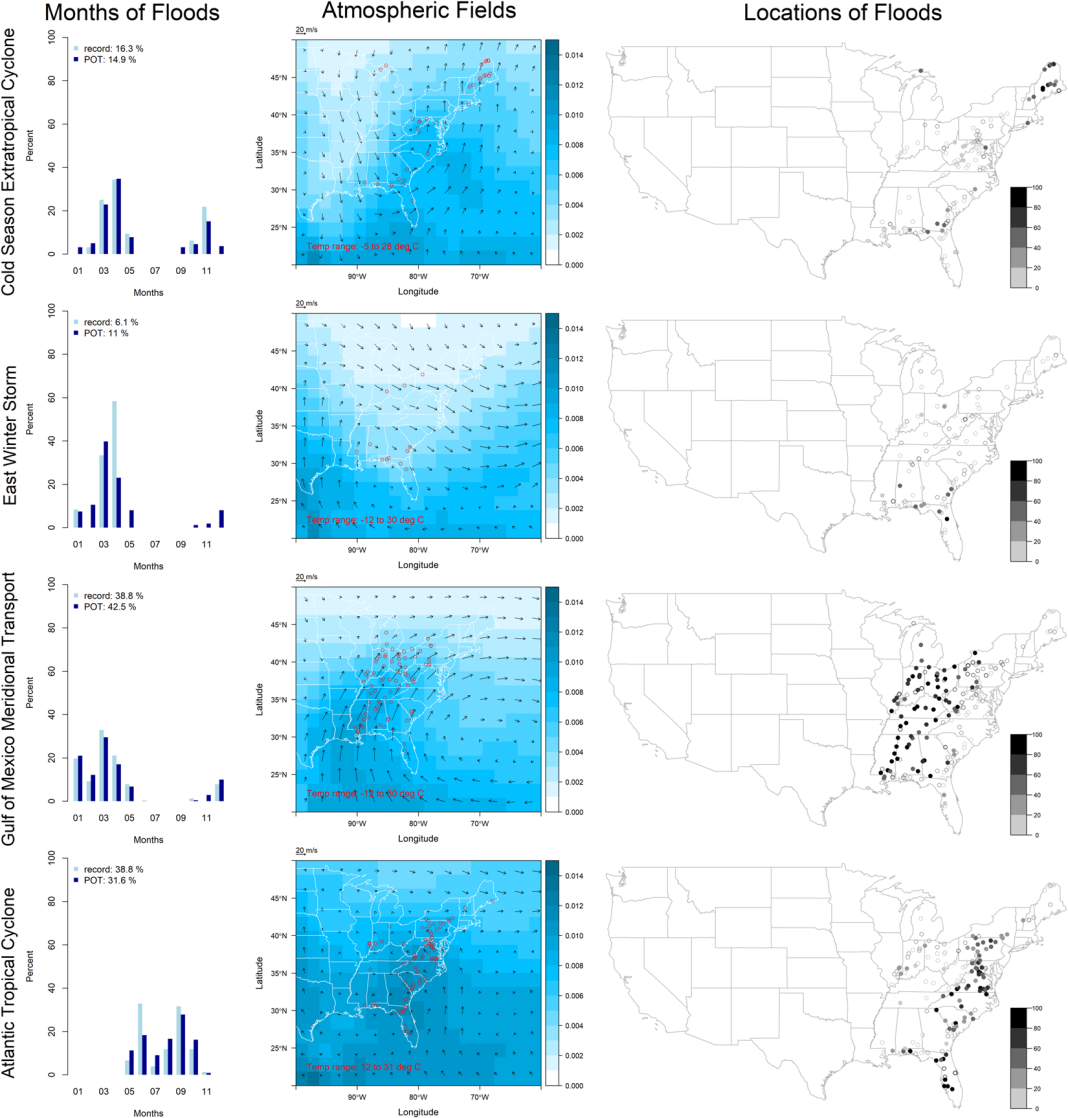
Circulation patterns in the East region (see Fig. 1 for explanation).

The East region (Fig. 3, Table 3) is represented by a 2 × 2 SOM which includes gages in HUCs 1 through 3, HUCs 5 and 6, and all gages in HUC 4 east of 87°W, for a total of 196 record floods and 1,467 POT floods. The East region has a longitude-latitude domain of 20°–50°N and 100°–60°W for the atmospheric fields. East region circulation patterns are *cold season extratropical cyclone*, *east winter storm*, *Gulf of Mexico meridional transport*, and *Atlantic tropical cyclone* (includes hurricanes).

**Table 3:** Circulation patterns in the East region.

SOM Code Number	Pattern Name (Abbreviation)	Number Record (POT) Floods	Timing	Atmospheric Signature	Primary Location of Influence	Localized Mechanisms
1	Cold Season Extratropical Cyclone (Cold_ETC)	32 (219)	Oct–Nov, Mar–May	Cold temperatures, centered trough pattern in the winds, low (high) specific humidity to the west (east) of the trough^62^	Far north, far south, and along eastern seaboard	Heavy rainfall, may be augmented by rain-on-snow^63,64^
2	East Winter Storm (East_WS)	12 (161)	Dec–May	Cold temperatures, weak cyclonic curvature in the winds in the northeast, very low specific humidity from the north	The south and the Appalachian Mountains	Heavy rainfall^65^
3	Gulf of Mexico Meridional Transport (east) (GofM_MT(e))	76 (623)	Dec–May	Cold temperatures, strong southerly winds from the Gulf of Mexico confined to a narrow region and steered into the Ohio River basin by the Bermuda High at 30°N and 70°W, relatively high specific humidity, evidence of a tropical moisture export^16,58.60,61^	West of the Appalachian Mountains	Heavy rainfall, may be augmented by rain-on-snow^66,67^
4	Atlantic Tropical Cyclone (Atlantic_TC)	76 (464)	May–Oct	Warm temperatures, weak cyclonic pattern over the Mid-Atlantic coast (due to averaging over multiple non-co-located cyclone centers), high specific humidity^68^	Eastern Appalachian Mountains and eastern seaboard	Heavy rainfall^23,69^

For a given circulation pattern, the record and POT floods generally occur at the same time of year and in the same geographical region; additionally, for a given pattern, the percent of all record and POT floods in the region that are assigned to the pattern is very similar. For example, in the West region, 43.2% and 47% of record and POT floods, respectively, are assigned to *snowmelt* and occur primarily in May and June in the Rocky Mountains (Fig. 1, top row). However, POT floods do span a wider range of months and a larger geographical region than record floods.

Some circulation patterns are evident in multiple regions. For example, the seasonality and atmospheric fields associated with *central* and *east winter storm* are very similar, especially in the north (for the portion of the Central atmospheric domain which overlaps with the East domain). Another example is *Gulf of Mexico meridional transport* which causes extreme flooding in both the Central and East regions in a continuous geographical domain stretching from the lower Mississippi River basin into the Ohio River basin.

Interestingly, some circulation patterns have similar atmospheric signatures but are displaced in time or space. For example, in the West region, *northern* and *southern pineapple express* are both cold season tropical moisture exports from the central Pacific, but the former is steered to the north by the North Pacific High. In the Central region, winds in both *warm season Great Plains jet* and *Gulf of Mexico meridional transport* come from the Gulf of Mexico and curve to the northeast, but the primary distinguishing characteristic between the two patterns is their seasonality, with associated differences in temperature and magnitude of moisture transport, as well as steering (*Gulf of Mexico meridional transport* is steered further eastward). In the East region, the *eastern winter storm*, which is often associated with a multi-day rising limb of the flood hydrograph, may be a *cold season extratropical cyclone* that has dissipated and moved to the northeast.

### Record and POT flood characteristics by circulation pattern

**Figure 4:**
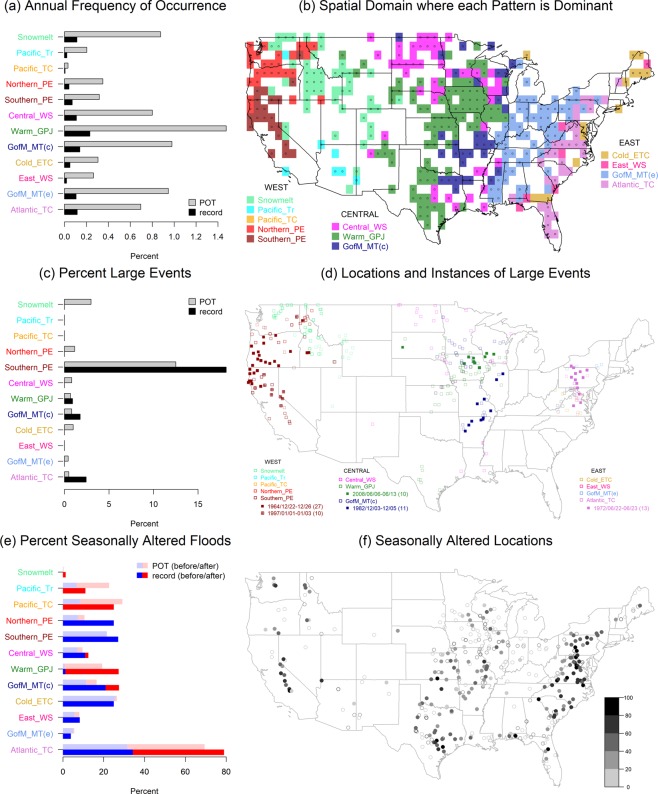
Flood characteristics by circulation pattern. (**a**) Annual frequency of flood occurrence (expressed as percent of gages associated with that pattern relative to all reporting gages in the United States at time of flood). (**b**) The most prominent pattern for each grid cell (white indicates no gages in that grid cell, and a dot indicates at least 50% of POT floods were caused by that pattern). (**c**) The percent of events which are large relative to all events associated with that pattern. (**d**) Locations of floods associated with large events (filled and empty symbols indicate record and POT floods, respectively, and for record large floods, the date range and number of affected gages are also provided). (**e**) The percent of seasonally altered floods relative to all floods associated with that pattern. (**f**) The location of seasonally altered floods (dots and circles indicate record and non-record POT floods, respectively, and the color bar indicates the percent of seasonally altered POT floods).

Figure 4 shows the characteristics of record and POT floods associated with each circulation pattern. *Warm season Great Plains jet* is the most active pattern, causing on average approximately 1.4% of all contiguous United States gages to have a POT flood in any given year. Other active patterns include *snowmelt* in the West region, *central winter storm* in the Central region, *Atlantic tropical cyclone* in the East region, and *Gulf of Mexico meridional transport* in the Central and East regions.

In the West region, the *northern* and *southern pineapple express* are the dominant circulation patterns on the coast and in the Sierra Nevada and Cascade Mountain ranges, *snowmelt* is the dominant pattern in the Rocky Mountains, and *Pacific trough* is the dominant pattern in the Southwest; *Pacific tropical cyclone* is never dominant. In the Central region, dominance of a single circulation pattern over a distinct spatial domain is not as evident as in the West region; however, *central winter storm* is generally confined to either far north or far south, while *warm season Great Plains jet* and *Gulf of Mexico meridional transport* are generally more active in the west and the east, respectively. In the East region, *Gulf of Mexico meridional transport* is clearly dominant in the Ohio River basin and southward; the remaining three circulation patterns impact the Atlantic seaboard but have no clear dominance over a given spatial domain.

Many record and POT floods caused by *southern pineapple express* occur in large events (i.e., multiple gage locations are flooded at the same time) and are primarily located in northern California and Oregon; particularly exceptional events occurred during Christmas 1964 and New Year’s 1997^20,21^. Other patterns that cause large events include *snowmelt* in the Rocky Mountains, *warm season Great Plains jet* and *Gulf of Mexico meridional transport* in the Midwest, particularly in December 1982 and June 2008^15,22^, and *Atlantic tropical cyclone* in the mid-Atlantic states, particularly Hurricane Agnes in June 1972^23^.

In the West region, altered flood seasonality (i.e., the record or POT flood occurs more than 90 days away from the mean day of flood occurrence) is most prominent in the Sierra Nevada Mountains, where nearly all record and POT floods occur in winter due to the *southern pineapple express*, rather than in the late spring due to snowmelt. Notably, seasonality is rarely altered in the Rocky Mountains and northern central United States where floods are caused by *snowmelt* or *central winter storm*. In the Central region, both *warm season Great Plains jet* and *Gulf of Mexico meridional transport* cause altered seasonality, most prominently in the lower western Mississippi River basin. In the East region, most record and POT floods east of the Appalachian Mountains caused by *Atlantic tropical cyclone* have an altered seasonality, occurring in late summer rather than during spring storms.

### Results outside the contiguous United States

Due to limited data availability, only a partial analysis was performed for HUCs 19 through 21 (for more details, see Supplemental Material). Alaska (HUC 19) is represented by a 2 × 2 SOM with a total of 17 record and 82 POT floods; the longitude-latitude domain is 54°–74°N and 170°–128°W for the atmospheric fields. Alaska circulation patterns are *Gulf of Alaska low*, *snowmelt*, *Bering Sea jet*, and *Gulf of Alaska jet*. Hawaii (HUC 20 without gages in Guam or American Samoa, which were not analyzed) is represented by a 2 × 2 SOM with a total of 27 record and 181 POT floods; the longitude-latitude domain is 10°–30°N and 170°–142°W for the atmospheric fields. Hawaii circulation patterns are *Pacific tropical cyclone*, *kona low*, *upper trough*, and *cold front*. Puerto Rico (HUC 21) is represented by a 1 × 2 SOM with a total of 12 record and 70 POT floods; the longitude-latitude domain is 10°–26°N and 74°–58°W for the atmospheric fields. Puerto Rico circulation patterns are *Atlantic tropical cyclone* and *cold front*.

### Limitations

One limitation of our analysis is the temporal and spatial coverage of the streamflow data. There are limited gage locations in the Southwest northward to Montana (Fig. 4) and there is a relatively high density of gages in the Central region (not shown). Additionally, because the longest available continuous water year record was used for each gage location, the data records are not consistent across locations. A potential consequence of this is limited characterization of circulation patterns in the Southwest; thus, it is possible that *Pacific tropical cyclone* would be dominant if more data were available. Furthermore, the frequency of flood events caused by circulation patterns in the Central region (e.g., *warm season Great Plains jet*), may be unduly enhanced due to the higher sampling.

Another limitation is the finite number of circulation patterns identified by the SOMs (i.e., 12 for the contiguous United States). Thus, a pattern which occurs infrequently may not be represented by a unique cluster. For example, in the West region, a few POT summer floods in the central Sierra Nevada Mountains and the Olympic Peninsula are assigned to *Pacific tropical cyclone*. Similarly, in the Central region, the few summer floods associated with tropical cyclones from the Gulf of Mexico or with mesoscale convective systems are assigned to *warm season Great Plains jet*. And finally, in the East region, some floods associated with warm season southerly moisture transport from the Gulf of Mexico are assigned to *Atlantic tropical cyclone*. In all these cases, the cluster assignments likely occur because *Pacific* or *Atlantic tropical cyclone* and *warm season Great Plains jet* are the only warm season circulation patterns in the West, East, and Central regions, respectively. A similar limitation is that only atmospheric fields are used as input data to the SOMs, meaning that antecedent conditions (e.g., snowpack and soil moisture) are not explicitly considered. This limitation can lead to incorrect cluster assignments for snowmelt or rain-on-snow floods and floods caused by light or normal rainfall on high soil moisture conditions. For example, the Central region has no snowmelt-only pattern despite snowmelt floods occurring in the northern portion of the region.

Finally, the SOMs as currently configured do not track the movement of an atmospheric phenomenon in space and time. This limitation is exacerbated by the fact that (i) the boundaries of the West, Central and East region are, to some degree, arbitrary discontinuities imposed on a continuous geographical domain, and (ii) the date of the flood is not explicitly provided as input data, but rather is implicit in that the atmospheric fields are from two days before the record or POT flood (the temperature data also implicitly indicates cold or warm season). In consequence, there is a limit to the extent a single atmospheric phenomenon can be transposed in space but still be identified as one phenomenon by the SOM. This is likely a contributing factor to the omission of Gulf of Mexico tropical cyclones in the Central region and is likely the reason why winds in *Atlantic tropical cyclone* in the East region are weak, having been smeared over multiple storm centers.

Another consequence of this final limitation is that the same circulation patterns affect multiple regions (e.g., *Gulf of Mexico meridional transport* in the Central and East regions) and one event may be split across regions or across patterns, which would negatively bias the calculation of large events and lead to incorrect cluster assignments. For example, in the East region, four locations in relatively close geographical proximity in Georgia and Florida experienced a record flood between April 2^nd^ and 5^th^ in 1948 but were assigned to *Gulf of Mexico meridional transport*, *cold season extratropical cyclone*, and *eastern winter storm* depending on the date. The specific atmospheric fields associated with each date (not shown) clearly indicate that all four record floods were caused by one atmospheric wave. However, that atmospheric wave changed sufficiently and quickly during its eastward progression across the United States such that the SOM assigned the floods to three different patterns. This limitation is likely amplified by the choice of atmospheric data only on the date two days before the flood because in general, daily circulation patterns exhibit large variability; however, the circulation patterns associated with extreme floods tend to be more persistent than usual (e.g., a front becomes stalled over a region). One possible solution would be to use a multi-day average of the atmospheric data, recognizing that doing so would cause spatiotemporal smearing of circulation patterns, possibly obscuring extremes.

## Discussion

This paper presents the first near-continental scale identification of synoptic-scale atmospheric circulation patterns associated with extreme floods. Furthermore, this study demonstrates that, despite some limitations, identification of these patterns can be effectively accomplished by applying SOMs at judiciously chosen spatial scales to basic reanalysis atmospheric fields associated with extreme floods (see methods for more details). The results of this analysis provide valuable insights that can be used for improved flood risk preparation and management.

One insight is that although 12 circulation patterns were identified for the contiguous United States, the similarity in atmospheric fields implies four primary categories: tropical moisture exports (encompasses *northern* and *southern pineapple express*, *warm season Great Plains jet*, and *Gulf of Mexico meridional transport* in both the Central and East regions; accounts for a total of 411 record and 2,782 POT floods), tropical cyclones (encompasses *Pacific tropical cyclone* and *Atlantic tropical cyclone*; accounts for a total of 80 record and 488 POT floods), atmospheric lows or troughs (encompasses *Pacific trough*, *eastern winter storm* and possibly *central winter storm*, and *cold season extratropical cyclone*; accounts for a total of 117 record and 1,028 POT floods) and melting snow (*snowmelt*; accounts for a total of 73 record and 575 POT floods; note that during cold season tropical moisture exports or atmospheric lows, melting snow can also be an important factor in causing floods at high elevations or northern locations). This result implies that overall there are only a few distinct types of circulation patterns causing extreme floods in the contiguous United States and highlights that these types of patterns should be prioritized in efforts to assess and improve weather forecasting models.

Another insight is gained in the identification of circulation patterns which tend to cause clustering of extreme floods and the associated spatial domains where clustering occurs. Broadly, the results show that large events usually occur in the West and Central regions due to tropical moisture exports, and in the East region due to tropical cyclones. This corroborates previous statistical analysis showing that record floods across the United States are “on average more extreme than would be expected if they occurred randomly, and that they tend to form spatial clusters”^24^. Additionally, regions which exhibit spatial clustering of record floods generally correspond to regions where the climatological length scale of extreme precipitation is high (i.e., extreme precipitation falls over a large region)^25^, although the correspondence is not exact. Knowledge of which regions tend to experience spatial clustering of extreme flood events enables improved disaster preparedness and management. For example, it may be possible to reduce the likelihood of uncontrolled spill from flood control reservoirs by coordinating operations to account for one large flood event that affects a whole region. Similarly, emergency supplies and essential facilities can be strategically placed to provide adequate aid in the scenario that a whole region is affected^26^.

A third insight is in understanding the dominant circulation patterns and seasonality of extreme floods in a given spatial domain. In a region dominated by one pattern, there is a high likelihood that extreme floods will only occur during the time of year the pattern occurs. Notably, this is not necessarily during the regular flood season, which can present significant challenges for flood control operations designed for regular season flood events. For example, in the Sierra Nevada Mountains, *southern pineapple express* causes rain-on-snow floods in December through March, which is several months before the regular snowmelt-driven flood season of May through June. In 1997 in particular, when a *southern pineapple express* followed an already abnormally wet winter, multiple levees failed and multiple reservoirs in the San Joaquin and Sacramento River basins were at capacity, threatening major downstream flooding; of these, the Don Pedro reservoir had an uncontrolled release of water over the emergency spillway^27^. Yet based on historical records, the Christmas Eve 1861 event, caused by a prolonged *southern pineapple express*, was even larger and more devastating^28^. Conversely, in a region where no one circulation pattern is dominant, there is the possibility of a much wider timeframe in which extreme floods may occur and furthermore, multiple extreme flood events may occur in the same year due to different patterns. For example, the Atlantic seaboard may experience floods throughout the whole year; in particular on the Pigg River in Virginia, the record flood occurred in September 1987 due to *Atlantic tropical cyclone* and the second highest flood occurred earlier that year in April due to *Gulf of Mexico meridional transport*. These results highlight regions of the country that would benefit from flood control and disaster preparedness operations designed for both the regular flood season and an extreme flood season that may occur at a different time of year.

There are a variety of directions to extend these results. The methodology could be improved to enable tracking of atmospheric wave propagation in space and time, enabling deeper understanding of whether one atmospheric phenomenon can engender multiple flood-causing circulation patterns. This could be accomplished by incorporating lagged atmospheric fields in the SOM. This method could be applied to other continents to develop a global understanding of circulation patterns associated with extreme floods. Further investigation could also examine the implications of these results for flood risk, including: dependence (are there dependencies between different circulation patterns that may lead to multiple temporally-proximate extreme floods), attribution (how does the frequency and intensity of the circulation patterns relate to large-scale oceanic-atmospheric modes of climate variability), forecasting (how often does the occurrence of a given circulation pattern result in an extreme flood), non-stationarity and projection (has the frequency and intensity of the circulation patterns changed historically and is it projected to change under climate change), and design (how can financial instruments and infrastructure be designed and operated for flood risk mitigation given an improved knowledge of the circulation patterns associated with extreme floods).

Finally, to further inform flood management for a particular location of interest and recognizing the limitations of the results presented herein, an interactive website with detailed information for each record flood has been developed (https://kschlef.shinyapps.io/ExtremeFloods/). In addition to the reanalysis data and SOM results, data on the presence and type of extreme precipitation^29^, tropical cyclone tracks^30^, and tropical moisture exports^31^, as well as information from historical reports, are provided where relevant and available. This additional data enables a more nuanced perspective on the natural causes of record floods. In particular, the information from historical reports provides insight into the importance of antecedent conditions in setting up the hydrologic conditions that enable an extreme flood. This website is a valuable resource that enables further understanding of the causes and characteristics of the record flood at a location of interest.

## Methods

### Data

Daily reanalysis atmospheric data was obtained from the 20^th^ Century V2 reanalysis data^32–34^, available from 1851–2014 at 2° grid resolution. The atmospheric fields used for analysis were, at the 850 mb level, specific humidity, omega (vertical velocity in pressure units), and zonal and meridional winds, and at the 1,000 mb level, temperature. Initial analysis only included the four atmospheric fields at 850 mb. Omega was chosen to provide information about convective processes, specific humidity and winds were chosen to provide information about the advection and availability of atmospheric moisture, and 850 mb, generally the top of the planetary boundary layer, was chosen as indicative of precipitation-producing processes occurring in the lower atmosphere. However, these atmospheric fields alone were not able to appropriately distinguish between cold- and warm-season circulation patterns; adding temperature at the 1,000 mb (i.e., near surface) level solved this problem.

Daily streamflow data was obtained from United States Geological Survey gages designated as part of the Hydro-Climatic Data Network^35^ for the contiguous United States (i.e., HUCs 1 through 18). Data pre-processing occurred as follows: gages with basin area less than 200 square miles were excluded; for each gage, after the exclusion of any data after 2014 (due to the availability of the reanalysis data), the longest period of continuous data was cut to begin and end with the water year (October 1 through September 30); any gages with less than 30 water years of continuous data were excluded. This process resulted in a total of 681 gages with data spanning water years 1874–2014. Gages in HUCs 19 (Alaska), 20 (Hawaii, Guam and American Samoa), and 21 (Puerto Rico) were processed separately following the same procedure except the requirement of a minimum basin area of 200 square miles was relaxed due to the limited number of available gages.

From the daily streamflow data, the record flood, the water year timeseries of annual maximum daily streamflow (AMS), and peaks-over-threshold (POT) floods were calculated for each gage. The record flood was defined to occur on the date associated with the maximum value of daily streamflow. The POT floods, of which the record floods are a subset, were defined to occur on the date associated with a local maxima value of daily streamflow over a threshold value. The threshold was set to the 10-year flood, determined by fitting a log-Pearson type III distribution to the AMS timeseries. Additionally, the POT floods were constrained to be at least eight days apart, to avoid double-counting an event. Due to the procedure used for data pre-processing, it is possible that the identified record flood is not the same as the known flood of record for a given location.

### Training and validation of clustering algorithm

Self-organizing maps (SOMs) were used to perform unsupervised clustering of the circulation patterns associated with floods. A SOM is an artificial neural network which uses a decaying neighborhood function and learning rate parameter to identify a one- or two-dimensional lattice of explanatory *codes* to which each individual data point is assigned^36^. The dimensions of the lattice determine the total number of codes as well as the number of neighbors for each code; here, a *2* × *3 SOM* indicates a lattice of two columns and three rows, with a total of six codes. SOMs were chosen over supervised manual clustering, which has been used for precipitation extremes^29^, because of reduced subjectivity and the ability to perform validation and easily extend to other datasets besides that used for training; similarly, SOMs were chosen over other clustering algorithms such as k-means or principal component analysis because they allow increased flexibility in cluster characteristics, they imply a topographical continuity between codes as imposed by the neighborhood function (although this can impose constraints on the number of codes), and they allow inclusion of multiple distinct input variables^37^. Additionally, SOMs have been previously used for clustering atmospheric circulation patterns associated with temperature and precipitation extremes: examples include Alaska^38^, the northwestern United States^39^, and central Italy^40^.

The input data to the SOM are the five reanalysis fields over a longitude-latitude domain for the date two days prior to each record or POT flood. As in previous similar studies, data in each grid cell was normalized (e.g., subtract the mean and divide by the standard deviation over all flood dates) and weighted by the square root of the cosine of latitude^39^. Only record floods were used to develop the SOMs; POT floods were subsequently assigned to existing clusters. Each code from the fitted SOMs represents a distinct pattern associated with the floods placed in that code’s cluster. The observed similarities between record and POT floods (discussed in results), provides further confidence in the SOMs in addition to the validation procedures (described below).

The key choices in developing the SOMs included (i) choice of region, specified by the flood locations and the longitude-latitude domain for the reanalysis data, (ii) choice of floods to use for initialization, and (iii) choice of map topography, specified by the number of codes and the dimensions of the rectangular lattice. A variety of different SOM configurations were assessed based on physical interpretability and robustness under cross-validation (described below and in further detail in Supplemental Material); the best SOMs were retained for further analysis.

Physical interpretability was assessed based on a three factors: (i) how well months of flood occurrence were partitioned (e.g., October floods should not be clustered with January floods), (ii) whether the code was a recognizable atmospheric pattern appropriate to the associated months (e.g., a code with wind fields that clearly show a hurricane pattern should be associated with floods which occur in late summer), and (iii) whether floods for which the atmospheric circulation pattern is known *a priori*, based on literature or historic reports or characteristics of the flood or streamflow timeseries, were correctly clustered (e.g., floods caused by a hurricane should be clustered together and should not be clustered with floods caused by a winter storm). This analysis, which was iterative and made use of the additional information now available for each record flood on the website, also informed the pattern name given to each of the SOM codes.

Cross-validation for a given SOM configuration was performed by first fitting the SOM to the full data to create an *original* cluster assignment. Subsequently, 100 trials were performed in which, excepting the floods used for initialization, 90% of the floods were used as *training* data to fit a new SOM; the new SOM was then used to predict the cluster assignments of the remaining 10% of *validation* floods. Robustness under cross-validation was assessed using the adjusted rand index (ARI) and a metric of flood reassignment. The ARI measures the degree of overlap between two different sets of clusters; an ARI of zero indicates no overlap while an ARI of one indicates complete overlap. For each of the 100 trials, an ARI was calculated between (i) the clusters of the original data and the training data and (ii) the clusters of the original data and the training and validation data combined. Flood reassignment was calculated as the percent of floods for which the cross-validation cluster assignment was different than the original cluster assignment in at least 10% of the 100 trials. High ARIs and low flood reassignment indicated a robust SOM configuration.

### Calculation of flood characteristics

The prominence of circulation patterns in different regions of the United States was determined by finding which pattern caused the most POT floods in each 1° grid cell. For each pattern, a percent flood occurrence was calculated for each year by dividing the number of floods associated with that pattern by the number of all operational gages across all patterns; this accounts for the different number of operational gages each year. The frequency of flood occurrence was calculated as the mean of the percent flood occurrence across all years.

Flood events (i.e., the same circulation pattern is associated with flooding at the same time at multiple gage locations) were identified by grouping the floods associated with a given pattern by the date of occurrence, such that no more than two days separate each consecutive flood within an event. Event size was defined to be the number of floods associated with each event. Large events were defined to have an event size at least as large as the 99^th^ percentile of event size calculated from all events across all patterns; this does not account for the different number of operational gages each year.

Circular statistics^41^ were used to (i) calculate the mean day of flood occurrence and the seasonality index from the AMS timeseries and (ii) calculate the difference between the day of the record or POT flood and the mean day of flood occurrence. A flood was considered seasonally altered if it occurred more than 90 days before or after the mean day of flood occurrence.

## Supplementary information

Supplemental Material

## Data Availability

Processed data and code are available on Hydroshare (Katherine Schlef, Extreme Floods Code and Data); original data are available from cited sources.
