# Supplementary material for: Atmospheric Circulation Patterns Associated with Extreme United States Floods Identified via Machine Learning
Source: Sci Rep. 2019 May 9;9:7171. doi: 10.1038/s41598-019-43496-w (PMC6509142; doi:10.1038/s41598-019-43496-w)
Supplement: Supplementary file 1 — Supplemental Material [file 41598_2019_43496_MOESM1_ESM.docx]

**Atmospheric Circulation Patterns Associated with Extreme United States Floods Identified via Machine Learning**

**Authors:** Katherine E. Schlef, Hamid Moradkhani, Upmanu Lall

**Supplemental Material**

This supplemental material contains two sections. The first details the process of choosing the self-organizing map (SOM) configurations presented in the paper for the contiguous United States. The second section provides more details on the analysis and results for hydrologic unit codes (HUCs) 19 through 21. References cited in this supplemental material are provided at the end.

**SOM Selection for the Contiguous United States**

Figure 1 shows validation results for different SOM configurations. To develop the initial SOM configurations, we divided the United States into regions based on the HUC boundaries and on topography. We note that (i) references to atmospheric circulation patterns are more fully described in the main text (for ease of interpretation, the final pattern names are used here where possible, although at the time of this analysis, the final patterns were not fully determined), (ii) both the HUC and United States Geological Survey gage identification number are provided when referring to specific gages, and (iii) initialization gages were chosen to be those gages with atmospheric fields clearly identifiable as a specific circulation pattern, based on manual inspection.

We chose an initial *West* region to include HUCs 14 through 18, which comprises basins draining to the Pacific Ocean and the Great Basin, for a total of 153 gages. Using a latitude-longitude domain of 20-56^o^N and 150-96^o^W, we tested the following SOM configurations:

- *West 2x2*: 2x2 with initialization HUC18_11427000 (*southern pineapple express*), HUC17_13340600 (*northern pineapple express*), HUC17_13295500 (*snowmelt*), and HUC15_09384000 (*Pacific tropical cyclone*)
- *West 2x3a*: 2x3 with initialization same as *West 2x2* plus HUC17_13075000 (*Pacific trough*) and HUC17_12465000 (*southern pineapple express*)
- *West 2x3b*: 2x3 with same initialization as *West 2x3a* except HUC18_11382000 (December 1964 *southern pineapple express*) was substituted for HUC17_12465000

*West 2x3a* has higher adjusted rand index (ARI) than *West 2x3b* and *West 2x2*, although *West 2x2* has the lowest gage switching at 18.3% (compared to 24.8% and 37.9% for *West 2x3a and West 2x3b*, respectively). *West 2x2* results in southwest floods caused by *Pacific trough* being clustered with *snowmelt* floods in the Rocky Mountains; this issue is resolved in *West 2x3a.* However, *West 2x3a* has a cluster with only one gage (HUC17_12465000, used for initialization) whose code strongly resembles the other *southern pineapple express*. This indicates that only five codes are needed, so we tested the following SOM configuration:

- *West 1x5*: 1x5 with initialization same as *West 2x2* plus HUC17_13075000 (*Pacific trough*)

However, *West 1x5* has relatively poor ARI, high levels of gage switching (52.3%), and low physical interpretability. This likely occurs because the topographic mapping characteristics of SOMs requires some level of continuity between adjacent codes. Thus, in the 2x3 configuration, every code has at least three neighbors, whereas in the 1x5 configuration, a code has only one or two neighbors, and the first and fifth code are topographically very distant.

We chose an initial *East* region to include HUCs 1 through 3, which comprises basins draining east of the Appalachian Mountains and the South, for a total of 121 gages. Using a latitude-longitude domain of 20-50^o^N and 100-60^o^W. we tested the following SOM configurations:

- *East 1x3*: 1x3 with initialization HUC3_02320500 (*east winter storm*), HUC3_02196000 (*Atlantic tropical cyclone*), and HUC2_01646502 (*cold season extratropical cyclone*)
- *East 2x2*: 2x2 with same initialization as *East 1x3* plus HUC3_02456500 (*Gulf of Mexico meridional transport*)

*East 1x3* has slightly better ARI than *East 2x2*, although *East 2x2* ARI is still good. While *East 1x3* has less gage switching than *East 2x2* (23.1% versus 50.4%), *East 2x2* has a separate cluster for *east winter storm* floods; in *East 1x3* those floods get clustered with floods associated with either *Gulf of Mexico meridional transport* or with *cold season extratropical cyclone*.

Given the initial *West* and *East* region, we defined an initial *Central* region to include HUCs 4 through 13, for a total of 407 gages. Using a latitude-longitude domain of 22-52^o^N, 120-78^o^W, we tested the following SOM configurations:

- *Central 2x2*: 2x2 with initialization HUC11_07186000 (*warm season Great Plains jet*), HUC7_05570000 (*Gulf of Mexico meridional transport*), HUC10_06354500 (*central winter storm*), and HUC10_06218500 (*snowmelt*)
- *Central 2x3*: 2x3 with initialization same as *Central 2x2* plus HUC10_06914000 (hurricane in the Gulf of Mexico) and HUC5_03161000 (*Atlantic tropical cyclone*)

Both have poor ARI, relatively high gage switching (55.8% and 53.6% for *Central 2x2* and *Central 2x3*, respectively), and low physical interpretability. In particular, for *Central 2x2,* floods from *Atlantic tropical cyclone* or hurricanes in the Gulf of Mexico are not identified. For both *Central 2x2* and *Central 2x3, snowmelt* floods in the Rocky Mountains are clustered with all other summer floods.

To resolve this issue, we expanded the *West* region to include all HUC 10 gages at elevation higher than 4,000 ft to make a new *West+* region, for a total of 169 gages. A 2x3 SOM for *West+* using the same latitude-longitude domain and initialization as *West 2x3a* resulted in negligible changes in ARI or gage switching (25.4%) and appropriate clustering of high elevation HUC 10 gages with other Rocky Mountain *snowmelt* floods.

We also expanded the *East* region to include HUCs 5 and 6 as well as all HUC 4 gages east of 87^o^W to make a new *East+* region, for a total of 196 gages. For *East+* we tested 1x3 and 2x2 SOMs (with same latitude-longitude domain and initialization as *East*) and found that the ARI and gage switching improve relative to the *East* SOMs. Similar to the *East* SOMs, while both have good ARI and gage switching, *East+ 1x3* is slightly better than *East+ 2x2* (11.2% and 24.5%, respectively); however, *East+ 2x2* has a separate cluster for floods associated with *east winter storm* while *East+ 1x3* does not.

Given the *West+* and *East+* regions, we defined a new *Central-* region to include all HUC 4 gages west of 87^o^W, HUCs 7 through 9, all HUC 10 gages at elevation lower than 4,000 ft, and HUCs 11 through 13, for a total of 316 gages. Using a latitude-longitude domain of 24-50^o^N, 112-86^o^W, we tested the following SOM configurations:

- *Central- 1x3*: 1x3 with initialization HUC11_07186000 (*warm season Great Plains jet*), HUC7_05570000 (*Gulf of Mexico meridional transport*), and HUC10_06354500 (*central winter storm*)
- *Central- 2x2r*: 2x2 with a randomly chosen initialization of HUC4_04080000, HUC7_05479000, HUC9_05062000, and HUC7_05486490
- *Central- 2x2a*: 2x2 with initialization same as *Central- 1x3* plus HUC10_06914000 (hurricane in the Gulf of Mexico in HUC 10)
- *Central- 2x2b*: 2x2 with initialization same as *Central- 1x3* plus HUC12_08189500 (hurricane in the Gulf of Mexico in HUC 12)
- *Central- 2x3r*: 2x3 with a randomly chosen initialization of HUC4_04080000, HUC7_05479000, HUC9_05062000, HUC7_05486490, HUC12_08101000, and HUC10_06207500
- *Central- 2x3*: 2x3 with initialization same as *Central- 2x2* plus HUC8_07363500 (*central winter storm*) and HUC12_08189500 (hurricane in the Gulf of Mexico)

*Central- 1x3* has high ARI and low gage switching (15.2%). *Central- 2x2a* has similar ARI to *Central- 1x3*, with the exception of a few poorly performing trials; *Central- 2x2r* has slightly decreased ARI but better gage switching relative to *Central- 2x2a* (29.4% versus 49.7%, respectively). Relative to configurations with less clusters, *Central- 2x3* has degraded ARI and more gage switching (50%) but is better than *Central- 2x3r* which has low ARI and high gage switching (79.4%). *Central- 1x3* and the two configurations with random initialization do not have a separate cluster for floods caused by hurricanes; *Central- 2x2a* and *Central- 2x3* do have a hurricane cluster, but it only contains one gage (HUC10_06207500) even though some floods in HUCs 11 and 12 are caused by hurricanes; interestingly, when a different hurricane is used for initialization as in *Central- 2x2b* there is no hurricane cluster.

Given the results with *Central-,* we decided to try splitting *Central-* into a south and north region to test if the hurricanes would be more easily identified.

We defined a *Central-S* region to include HUCs 8 and 11 through 12, for a total of 117 gages. Using a latitude-longitude domain of 24-40^o^N and 110-86^o^W, we tested the following SOM configurations:

- *Central-S* *1x2*: 1x2 with initialization HUC11_07186000 (*warm season Great Plains jet*) and HUC8_07289500 (*Gulf of Mexico meridional transport*)
- *Central-S* *1x3*: 1x3 with initialization same as *Central-S* *1x2* plus HUC12_08189500 (hurricane in the Gulf of Mexico)
- *Central-S* *2x2*: 2x2 with initialization same as *Central-S* *1x3* plus HUC8_07363500 (*central winter storm*)

For the Central*-S* configurations, as the number of clusters increases, the gage switching increases (6%, 40.2%, and 72.6%, respectively) while the ARI, which is relatively low even for *Central-S* *1x2*, decreases; furthermore, none of the configurations place hurricanes in a separate cluster, instead grouping them into the cluster for *warm season Great Plains jet*.

We defined a *Central-N* region to include HUCs 7, 9, all gages in HUC 10 at elevation less than 4,000 ft, and all gages in HUC 4 west of 87^o^N, for a total of 199 gages. Using a latitude-longitude domain of 30-52^o^N and 114-84^o^W, we tested the following SOM configuration:

- *Central-N 1x3*: 1x3 with initialization HUC10_06817500 (*warm season Great Plains jet*), HUC7_05570000 (*Gulf of Mexico meridional transport*), and HUC10_06354500 (*central winter storm*)
- *Central-N 2x2*: 2x2 with initialization same as *Central-N 1x3* plus HUC10_06914000 (hurricane in the Gulf of Mexico)

Both *Central-N 1x3* and *Central-N 2x2* have relatively high ARI and low gage switching (19.6% and 22.1%, respectively); *Central-N 2x2* has a hurricane cluster while *Central-N 1x3* does not.

Based on this exploratory analysis, we accounted for both robustness and physical interpretability in choosing the final SOMs.

We chose *West+ 2x3* because it is very robust (high ARI and low gage switching) even with the inclusion of high elevation HUC 10 gages, which resolves the issue of inappropriate clustering of those gages when included in the *Central* region. Additionally, despite having a cluster with one gage whose code has the same physical interpretation as another cluster, it appropriately separates Rocky Mountain *snowmelt* floods from *Pacific trough* floods in the southwest, unlike the 2x2 configuration (which is also less robust).

We chose *East+ 2x2* because it also improves upon the robustness of *East 2x2* while resolving the issue of not capturing *Atlantic tropical cyclone* in the *Central* region. Additionally, while it has slightly lower ARI and slightly higher gage switching than *East+ 1x3*, both metrics are still satisfactory. It was deemed important to have a separate cluster for floods associated with *east winter storm*, which is also consistent with the clustering results for the *Central-* region.

We chose *Central- 1x3* because it was the most robust configuration for the *Central* region even though floods resulting from hurricanes in the Gulf of Mexico are clustered with *warm season Great Plains jet*. Even though *Central- 2x2a* is relatively robust and does include a hurricane cluster, that cluster only has one hurricane, which occurs in HUC 10, and hurricanes along the coast of Texas and Louisiana are still not captured. Thus, *Central- 1x3* was deemed a better option than *Central- 2x2a*.

With the insight gained from developing the above SOMs, we tested configurations for all HUCs in the contiguous United States (HUCs 1 through 18, for a total of 681 gages), using a latitude-longitude domain of 20-56^o^N and 150-60^o^W:

- *US 2x4r:* 2x4 with randomly chosen initialization HUC11_07095000, HUC10_06468170, HUC3_02058400, HUC5_03434500, HUC3_02313000, HUC10_06177500, HUC11_07257000, HUC10_06928000
- *US 2x4*: 2x4 with initialization HUC18_11427000 (*southern pineapple express*), HUC17_13340600 (*northern pineapple express*), HUC17_13295500 (*snowmelt*), HUC3_02196000 (*Atlantic tropical cyclone*), HUC2_01646502 (*cold season extratropical cyclone*), HUC3_02456500 (*Gulf of Mexico meridional transport*), HUC11_07186000 (*warm season Great Plains jet*), HUC10_06354500 (*central winter storm*)
- *US 3x3r*: 3x3 with randomly chosen initialization HUC7_05434500, HUC17_13336500, HUC11_07061500, HUC17_13317000, HUC3_02322500, HUC12_08109800, HUC5_03360500, HUC12_08086212, HUC17_14306500
- *US 3x3*: 3x3 with same initialization as *US 2x4* plus HUC17_13075000 (*Pacific trough*)
- *US 2x5r*: 2x5 with randomly chosen initialization HUC10_06334500, HUC17_13345000, HUC7_05567500, HUC12_08189500, HUC17_14321000, HUC4_04100500, HUC7_05317000, HUC10_06814000, HUC7_05405000, HUC2_01532000
- *US 2x5*: 2x5 with same initialization as *US 3x3* plus HUC15_09384000 (*Pacific tropical cyclone*)
- *US 3x4r*: 3x4 with randomly chosen initialization HUC12_08190500, HUC12_08171000, HUC10_06479000, HUC7_05434500, HUC11_07348000, HUC11_07196500, HUC10_06889500, HUC2_02018000, HUC10_06352000, HUC1_01031500, HUC7_05397500, HUC10_06446000
- *US 3x4*: 3x4 with same initialization as *US 2x5* plus HUC12_08189500 (hurricane in the Gulf of Mexico in HUC 12), HUC7_05570000 (*Gulf of Mexico meridional transport*)
- *US 2x7r*: 2x7 with randomly chosen initialization HUC17_14328000, HUC11_07151500, HUC7_05300000, HUC10_06878000, HUC7_05430500, HUC2_02030500, HUC3_02202500, HUC11_07095000, HUC7_05546500, HUC8_07290000, HUC10_06481500, HUC17_12401500, HUC1_01047000, HUC13_08289000
- *US 2x7*: 2x7 with same initialization as *US 3x4* plus HUC3_02320500 (*east winter storm*), HUC10_06914000 (hurricane in the Gulf of Mexico in HUC 10)
- *US 3x5r*: 3x5 with randomly chosen initialization HUC10_06446000, HUC10_06775900, HUC17_12413500, HUC3_02488500, HUC12_08053500, HUC10_06354500, HUC11_07261500, HUC11_07299670, HUC17_13295500, HUC2_01646502, HUC1_01022500, HUC4_04078500, HUC2_01644000, HUC17_12302055, HUC1_01064500
- *US 3x5*: 3x5 with same initialization as *US 2x7* plus HUC17_12465000 (*southern pineapple express*)
- *US 4x4r*: 4x4 with randomly chosen initialization HUC6_03465500, HUC13_08276500, HUC17_13139510, HUC18_11476500, HUC17_12307500, HUC10_06359500, HUC5_03167500, HUC3_02226000, HUC12_08146000, HUC10_06809500, HUC18_11469000, HUC12_08205500, HUC7_05455500, HUC11_07216500, HUC17_12452800, HUC11_07144780
- *US 4x4*: 4x4 with same initialization as *US 3x5* plus HUC10_06218500 (*snowmelt*)

For all *US* SOMs (i.e., those at the scale of the contiguous United States), we found low robustness and poor physical interpretability, regardless of the configuration or initialization. This result is in contrast to the high robustness and good physical interpretability of the regional SOMs described above. Thus, we chose the regional SOMs *West+ 2x3, Central- 1x3,* and *East+ 2x2* as the final configurations to present in the paper. For clarity and brevity, these final SOM choices are called simply *West*, *Central,* and *East* in the main text.

*
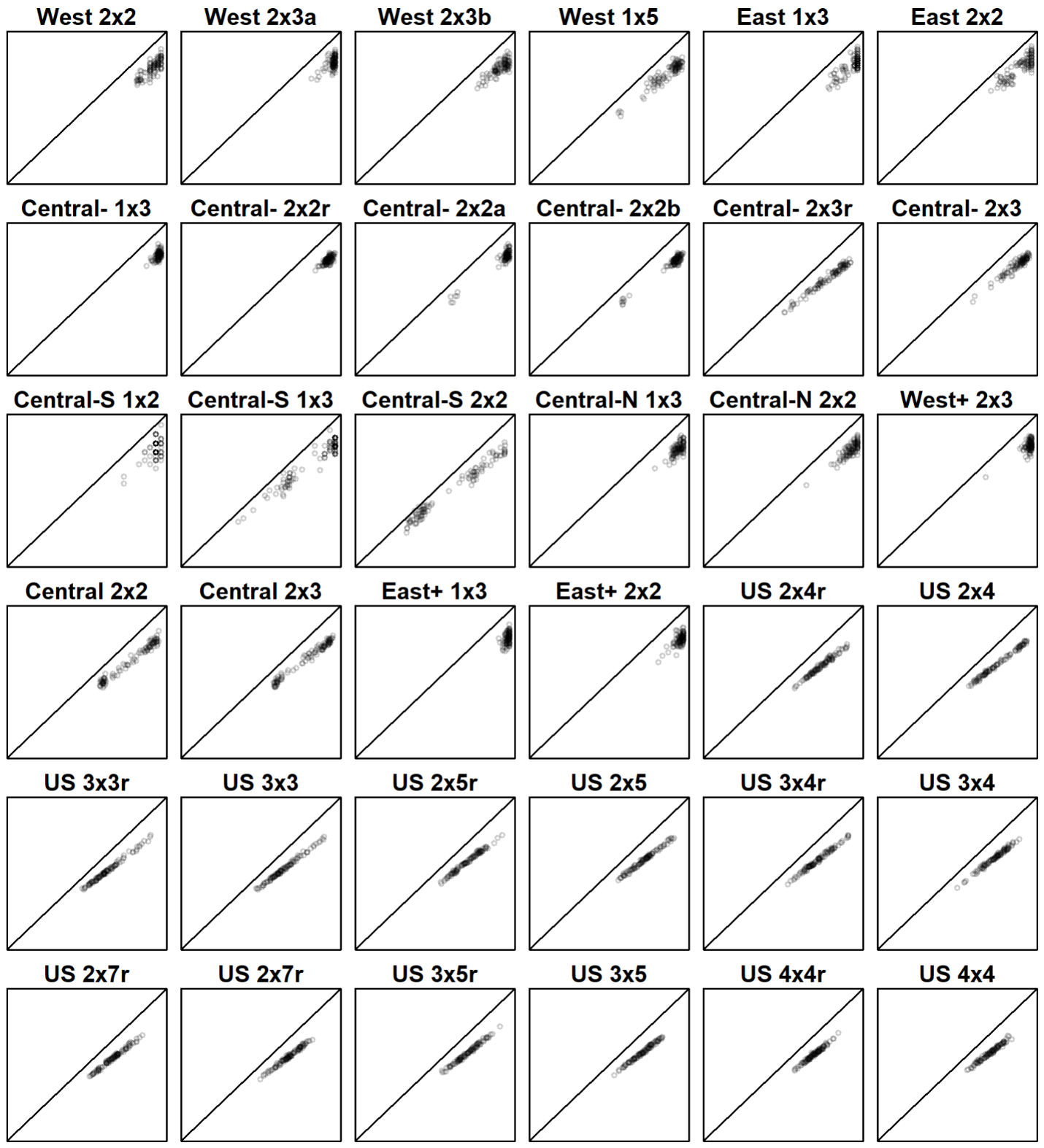
*

Figure 1: For each SOM configuration, indicated by the title, the points show the ARI of the training dataset (x-axis) and the full dataset (training plus calibration) (y-axis) relative to the original SOM for all 100 trials. All axes range from zero to one (lower left to top right, respectively) (axis numbers omitted for clarity); each plot has a one-to-one line.

**SOM Selection and Results for HUCs 19 through 21**

Figure 2 shows validation results for different SOM configurations for HUCs 19 through 21.

In Alaska (Figure 3, Table 1), there is limited streamflow information over a vast geographical area and many floods are caused by snowmelt, making the identification of atmospheric circulation patterns causing extreme floods particularly difficult^1^. From the historical reports associated with extreme floods, it is apparent that extreme floods in Alaska are caused by snowmelt and low-pressure systems which may be the remnants of a typhoon. Thus, we tested a 1x2 SOM with initialization of HUC19_15292000 (*snowmelt*) and HUC19_15290000 (*Gulf of Alaska jet*), a 1x3 SOM with initialization of HUC19_15292000 (*snowmelt*), HUC19_15484000 (*Bering Sea jet*), and HUC19_15022000 (*Gulf of Alaska intensified low*), and a 2x2 SOM with initialization same as the 1x3 SOM plus HUC19_15290000 (*Gulf of Alaska jet*). The 1x2 SOM had poor ARI and high gage switching; because *Gulf of Alaska intensified low* has such a strong and unique signal, it is assigned a cluster and all other floods are grouped together. Both the 1x3 and 2x2 SOMs had good ARI and low gage switching, however, the advantage of the 2x2 SOM is that *Bering Sea jet* and *Gulf of Alaska jet* are distinguished. Of the floods for which the circulation pattern is known based on historical reports, only one seems to be mis-assigned in the 1x3 and 2x2 SOMs; the Oct. 11^th^ 1986 record flood at HUC19_15292700 is assigned to snowmelt even though it was caused “by a low-pressure trough … that was blocked by a high-pressure ridge … the resultant storm front was stationary for 36 hours.”^2^

In Hawaii (Figure 4, Table 2), there are four synoptic-scale systems causing heavy rainfall that leads to floods: upper troughs, cold fronts from the mid-latitudes, kona lows, and tropical cyclones^3^. Thus, we chose a 2x2 SOM with initialization of HUC20_16019000 (*Pacific tropical cyclone*), HUC20_16304200 (*kona low*)*,* HUC20_16330000 (*cold front* in historical reports, but assigned to *upper trough* by the SOM) and HUC20_16071500 (*upper trough*). This 2x2 SOM has acceptable ARI and gage switching; a 1x3 SOM (same initialization except without HUC20_16071500) has better ARI but cannot distinguish *kona low* from *upper trough*. Note that even though Hawaii has the most available gages, compared to Alaska and Puerto Rico, there is still relatively few compared to the regions used for the contiguous United States; it may be possible, though not attempted here, to add a few more gages based on a recent analysis of which gages in Hawaii are unimpaired^4^.

For Puerto Rico (Figure 5, Table 3), analysis of the historical reports associated with the record floods indicate that all record floods were caused by *Atlantic tropical cyclone* except for the December 12^th^, 1987 record flood, which was caused by *cold front*. Thus, we chose a 1x2 SOM with initialization of HUC21_50063800 (*cold front*) and HUC21_50063800 (*Atlantic tropical cyclone*). This 1x2 SOM has good ARI and low gage switching. The limitation is that all cold season POT floods are assigned to *cold front* even though the actual circulation pattern may be different.


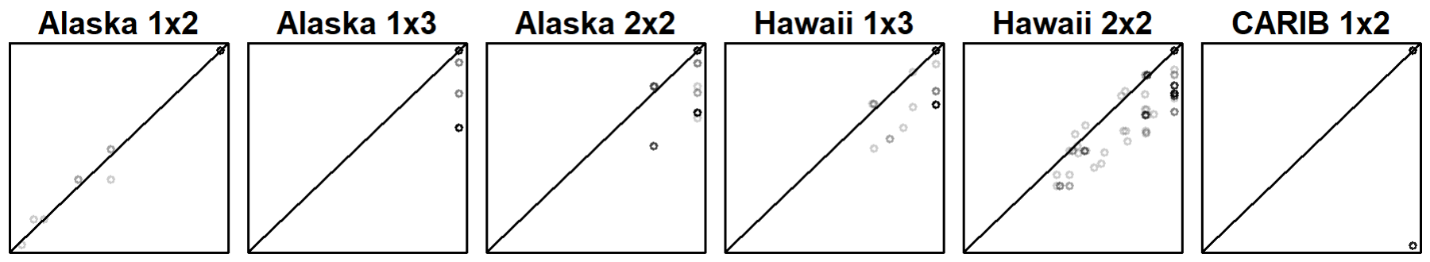


Figure 2: ARIs for the SOM configurations associated with HUCs 19 through 21 (where CARIB designates Puerto Rico). See Figure 1 for more explanation.


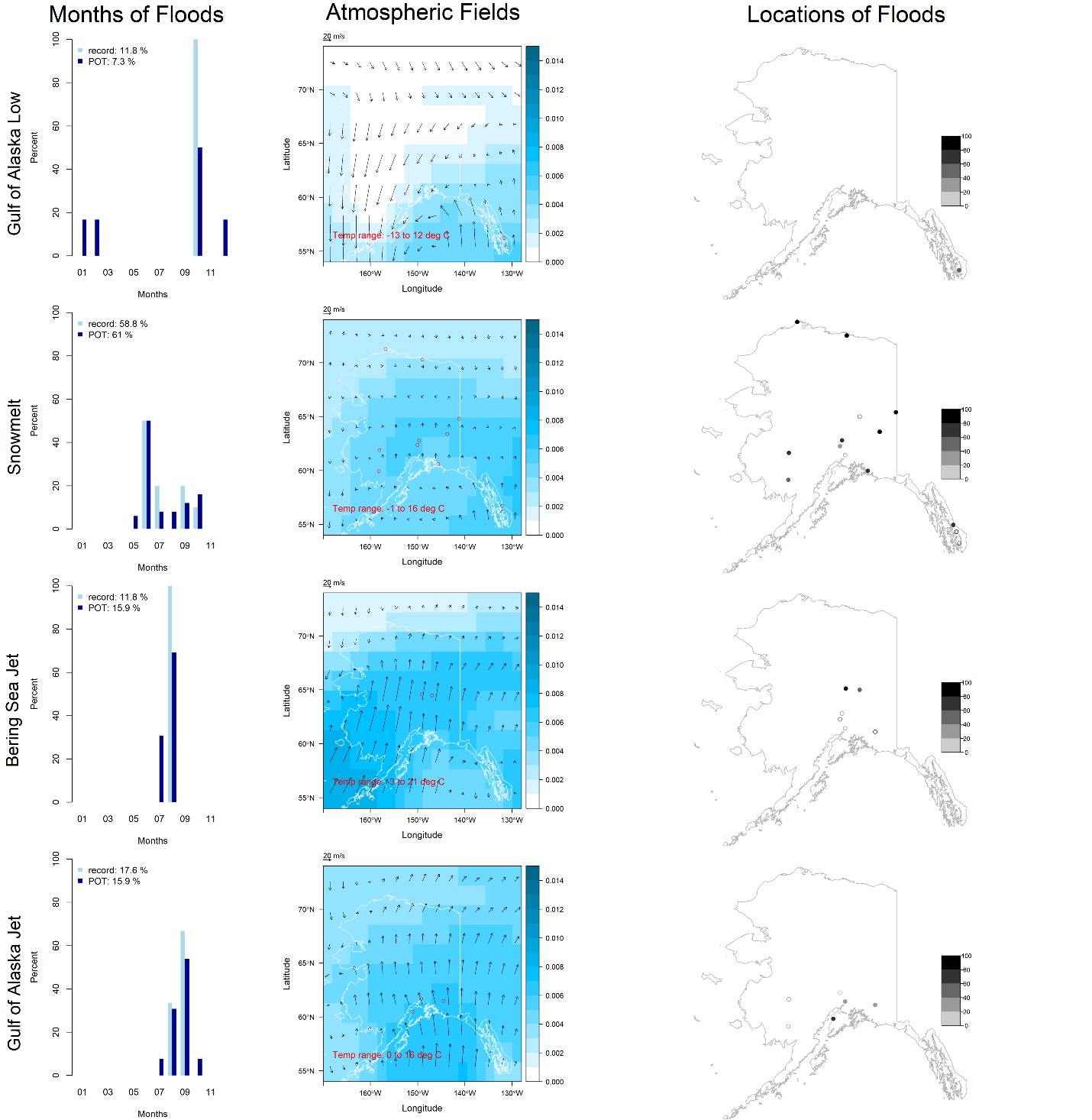


Figure 3: Circulation patterns in Alaska (HUC 19). Each row represents a unique pattern. Column one shows the percent of record or POT floods occurring in each month for that pattern (the legend provides the percent of record or POT floods assigned to that pattern relative to all patterns in the region). Column two shows the wind vectors (multiplied by two for plotting, units m/s) and the specific humidity field (units kg/kg, magnitude indicated by the color bar) representative of the cluster (the text indicates the temperature range, and the circles are locations of record floods in that cluster); for visualization purposes, the inverse of normalization was applied to the atmospheric fields obtained from the SOM. Column three shows the location of record and POT floods (dots and circles, respectively) in that cluster (the color bar indicates the percent of POT floods assigned to that cluster).


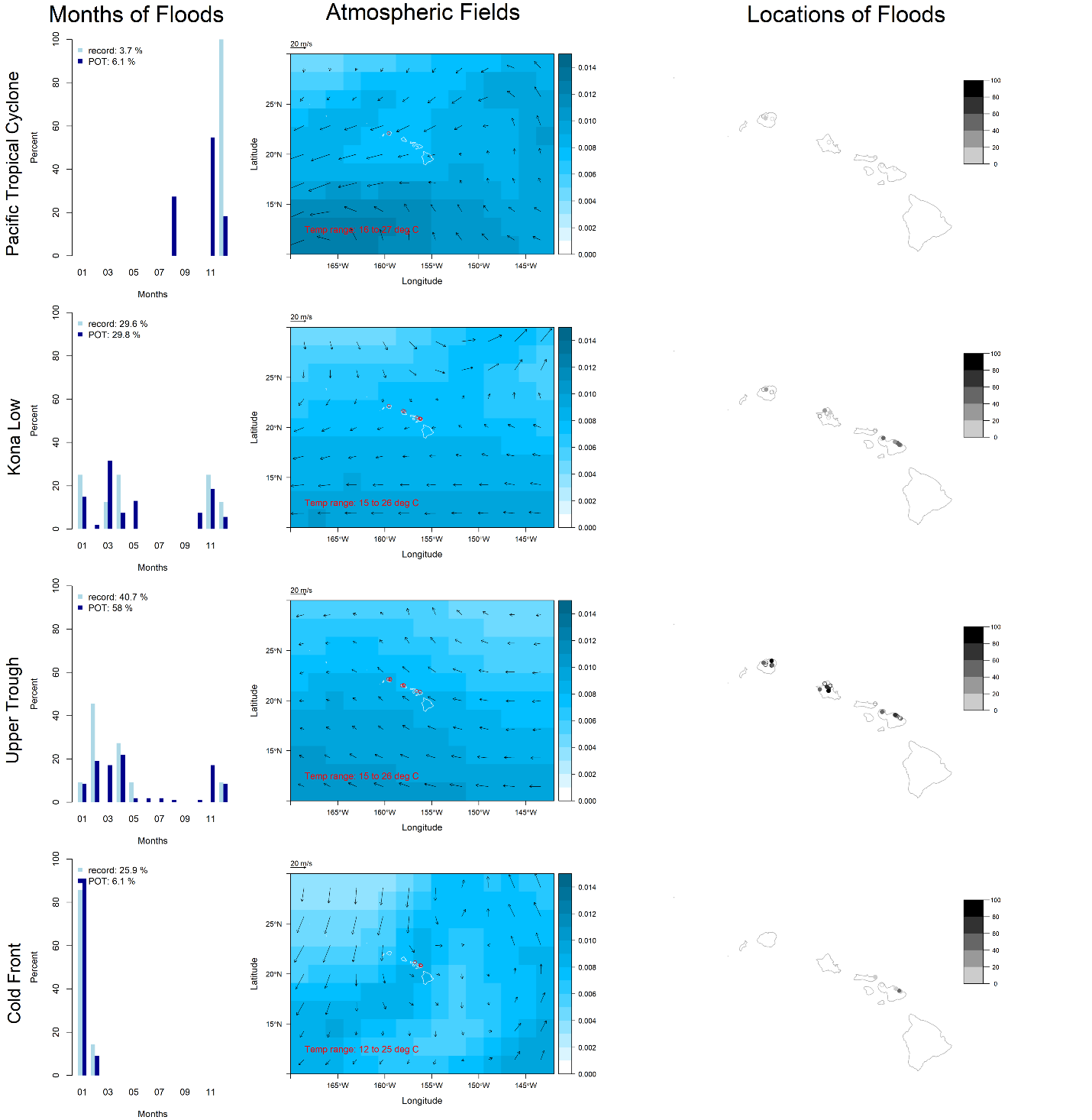


Figure 4: Circulation patterns in Hawaii (HUC 20 without gages in Guam or American Samoa) (see Figure 3 for explanation).


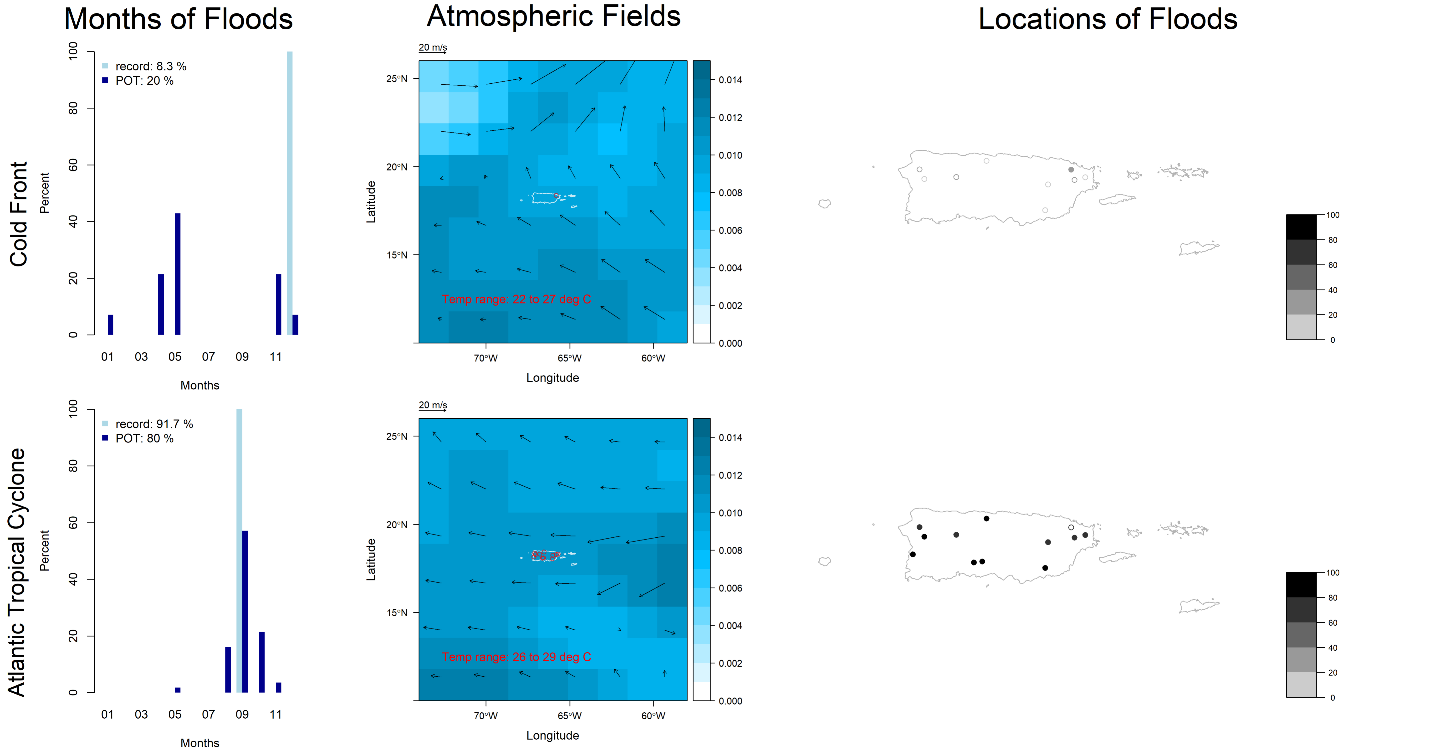


Figure 5: Circulation patterns in Puerto Rico (HUC 21) (see Figure 3 for explanation).

Table 1: Circulation patterns in Alaska (HUC 19).

| **SOM**  **Code Number** | **Pattern Name** | **Number Record (POT) Floods** | **Timing** | **Atmospheric Signature** | **Primary Location of Influence** | **Localized Mechanisms** |
| --- | --- | --- | --- | --- | --- | --- |
| 1 | Gulf of Alaska Low | 2 (6) | Oct-Feb | Cold temperatures, low pressure system in the Gulf of Alaska, very low specific humidity, strong northerly winds in the west^5^ | Southeast Alaska | Likely heavy rain |
| 2 | Snowmelt | 10 (50) | May-Oct | Very weak winds, relatively low specific humidity | Alaska | Snowmelt^6^ |
| 3 | Bering Sea Jet | 2 (13) | Jul-Aug | Strong southwesterly winds from the Bering sea with high specific humidity^7^ | Interior Alaska | Heavy rain^7^ |
| 4 | Gulf of Alaska Jet | 3 (13) | Jul-Oct | Strong southerly winds from the Gulf of Alaska with moderate specific humidity^8^ | South-Central Alaska | Heavy rain, possibly augmented by melting glaciers and snow^8^ |

Table 2: Circulation patterns in Hawaii (HUC 20 without gages in Guam or American Samoa).

| **SOM Number** | **Pattern Name** | **Number Record (POT) Floods** | **Timing** | **Atmospheric Signature** | **Primary Location of Influence** | **Localized Mechanisms** |
| --- | --- | --- | --- | --- | --- | --- |
| 1 | Pacific Tropical Cyclone | 1 (11) | Aug, Nov-Dec | Relatively warm temperatures, cyclonic winds and high specific humidity to the southwest^3^ | Hawaii | Heavy rain^9^ |
| 2 | Kona Low | 8 (54) | Oct-May | Strong easterly winds to the south, areas of high pressure to the northeast and northwest, low pressure directly north^3,10,11^ | Hawaii | Heavy rain^12^ |
| 3 | Upper Trough | 11 (105) | Nov-May | High pressure to the northeast that directs southeasterly winds, indication of a low to the west^3^ | Hawaii | Heavy rain^13^ |
| 4 | Cold Front | 7 (11) | Jan-Feb | Relatively cold temperatures, strongly northerly winds to the west, a distinct trough to the north, and cyclonic winds to the east^3^ | Hawaii | Heavy rain |

Table 3: Circulation patterns in Puerto Rico (HUC 21).

| **SOM Number** | **Pattern Name** | **Number Record (POT) Floods** | **Timing** | **Atmospheric Signature** | **Primary Location of Influence** | **Localized Mechanisms** |
| --- | --- | --- | --- | --- | --- | --- |
| 1 | Cold Front | 1 (14) | Nov-May | Relatively cooler temperatures, south-easterly winds, low pressure system in the northwest, high specific humidity in the southwest | Puerto Rico | Heavy rain^14^ |
| 2 | Atlantic Tropical Cyclone | 11 (56) | Aug-Oct | Relatively warmer temperatures, easterly winds, high specific humidity in the east | Puerto Rico | Heavy rain^15^ |

**References**

1. Perry, C. A., Aldridge, B. N. & Ross, H. C. *Summary of Significant Floods in the United States, Puerto Rico, and the Virgin Islands, 1970 through 1989*. (USGS Water-Supply Paper 2502, 2001). doi:10.1104/pp.111.180646

2. Lamke, R. & Bigelow, B. *Floods of October 1986 in Southcentral Alaska*. (USGS Open-File Report 87-391, 1988).

3. Schroeder, T. A. Meteorological Analysis of an Oahu Flood. *Mon. Weather Rev.* **105,** 458–468 (1977).

4. Clilverd, H. M., Tsang, Y.-P., Infante, D. M., Lynch, A. J. & Strauch, A. M. Long-term streamflow trends in Hawai‘i and implications for native stream fauna. *Hydrol. Process.* (2018). doi:10.1002/hyp.13356

5. O’Connor, J. F. The Weather and Circulation of October 1961. *Mon. Weather Rev.* **90,** (1962).

6. Meyer, D. F. *Flooding in the Middle Koyukuk River Basin, Alaska August 1994*. (USGS Water-Resources Investigations Report 95-4118, 1995).

7. Childers, J. M., Meckel, J. P. & Anderson, G. S. *Floods of August 1967 in East-Central Alaska*. (USGS Water-Supply Paper 1880-A, 1972).

8. National Weather Service. *Flood Report South Central Alaska Floods September 19 - October 2, 1995*. (1996).

9. Dunn, C. R. The Weather and Circulation of December 1957. *Mon. Weather Rev.* **85,** 409–416 (1957).

10. Simpson, R. H. Evolution of the Kona Storm, a Subtropical Cyclone. *J. Meteorol.* **9,** (1952).

11. Otkin, J. A. & Martin, J. E. A Synoptic Climatology of the Subtropical Kona Storm. *Mon. Weather Rev.* **132,** 1502–1517 (2004).

12. National Weather Service. High Winds and Flooding Rains December 10-14 2008. (2019). Available at: http://www.prh.noaa.gov/hnl/pages/events/WindFlood20081210/. (Accessed: 1st August 2019)

13. Posey, J. W. The Weather and Circulation of April 1963: Continued Warm East of the Continental Divide and Cool to the West. *Mon. Weather Rev.* **91,** 347–352 (1963).

14. McCabe, G. J., Barker, J. L. & Chase, E. B. Review of Water Year 1988 Hydrologic Conditions and Water-Related Events. in *National Water Summary 1988-89 - Hydrologic Events and Floods and Droughts* (eds. Paulson, R. W., Chase, E. B., Roberts, R. S. & Moody, D. W.) (USGS Water-Supply Paper 2375, 1991).

15. Torres-Sierra, H. *Hurricane Hortense: Impact on Surface Water in Puerto Rico*. (USGS Fact Sheet FS-014-97, 1997).
